# Simple indictor of increased blood culture contamination rate by detection of coagulase-negative staphylococci

**DOI:** 10.1038/s41598-021-96997-y

**Published:** 2021-09-02

**Authors:** Kei Yamamoto, Kazuhisa Mezaki, Norio Ohmagari

**Affiliations:** 1 Disease Control and Prevention Center/Travel Clinic, National Center for Global Health and Medicine, 1-21-1 Toyama, Shinjuku-ku, Tokyo, 162-8655 Japan; 2 Clinical Laboratory Department, National Center for Global Health and Medicine, 1-21-1 Toyama, Shinjuku-ku, Tokyo, 162-8655 Japan

**Keywords:** Clinical microbiology, Infectious-disease diagnostics

## Abstract

Coagulase-negative staphylococci (CoNS) are the most frequent contaminating bacteria; therefore, we aimed to investigate an indicator of CoNS to predict the increase in blood culture contamination rate (ConR). We performed a retrospective study of selected patients, who underwent blood culture testing. Contamination was defined as the presence of either one of two or more sets of skin-resident bacteria, except for cases with a low likelihood of contamination based on clinical aspects. We calculated the monthly ConR [(total number of contaminated cases per month)/(total number of blood culture sets collected per month) × 100] and analysed the ConR prediction ability using the following four indicators: the number of CoNS-positive sets of blood cultures, cases with at least one CoNS-positive blood culture set, cases with only one CoNS-positive blood culture set, and cases of contamination by CoNS. Cases with CoNS-positive blood cultures correlated with ConR (r = 0.85). Although the area under the receiver operating characteristic curve for the number of cases with ConR ≥ 2.5 differed significantly from that of the number of cases contaminated by CoNS, the negative predictive value was high, reaching up to 95.5% (95% confidential interval 87.3–99.1). The number of CoNS-positive cases could help predict an increase in ConR ≥ 2.5.

## Introduction

Relevant culture tests are important for the appropriate use of antimicrobial agents to combat antimicrobial-resistant bacteria. However, these tests can result in increased contamination, leading to an excessive use of antimicrobial agents, contributing to longer hospital stays and higher costs^1,2^. The blood culture contamination rate (ConR) is calculated retrospectively based on certain criteria^3^; however, its calculation is time-consuming and requires additional labour. Coagulase-negative staphylococci (CoNS) are the most frequently detected bacteria in blood culture contamination^2^. Therefore, we intended to investigate a simple real-time indicator of CoNS for predicting the increase in ConR. The values of the four indicators were aggregated as follows: the number of CoNS-positive sets of blood cultures, cases with at least one CoNS-positive blood culture set, cases with only one CoNS-positive blood culture set, and cases of contamination by CoNS.

## Results

**Figure 1:**
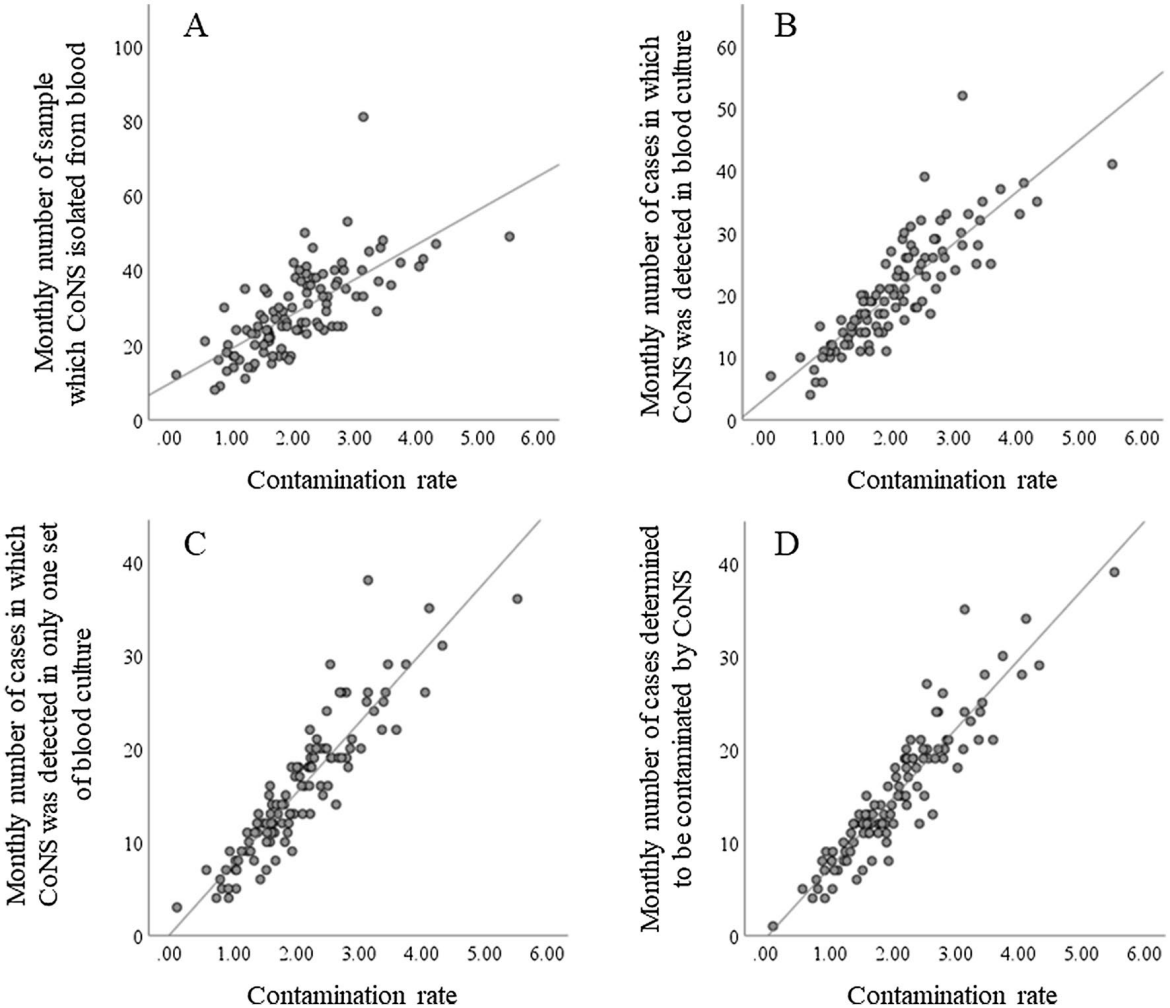
Scatter plot of correlation of ConR of each group (Indicator A–D). (**A**) Number of CoNS detected per specimen. (**B**) Number of CoNS detected per case. (**C**) Cases with only one set of positive CoNS. (**D**) Contamination cases by CoNS. *ConR* contamination rate, *CoNS* coagulase-negative staphylococci.

**Figure 2:**
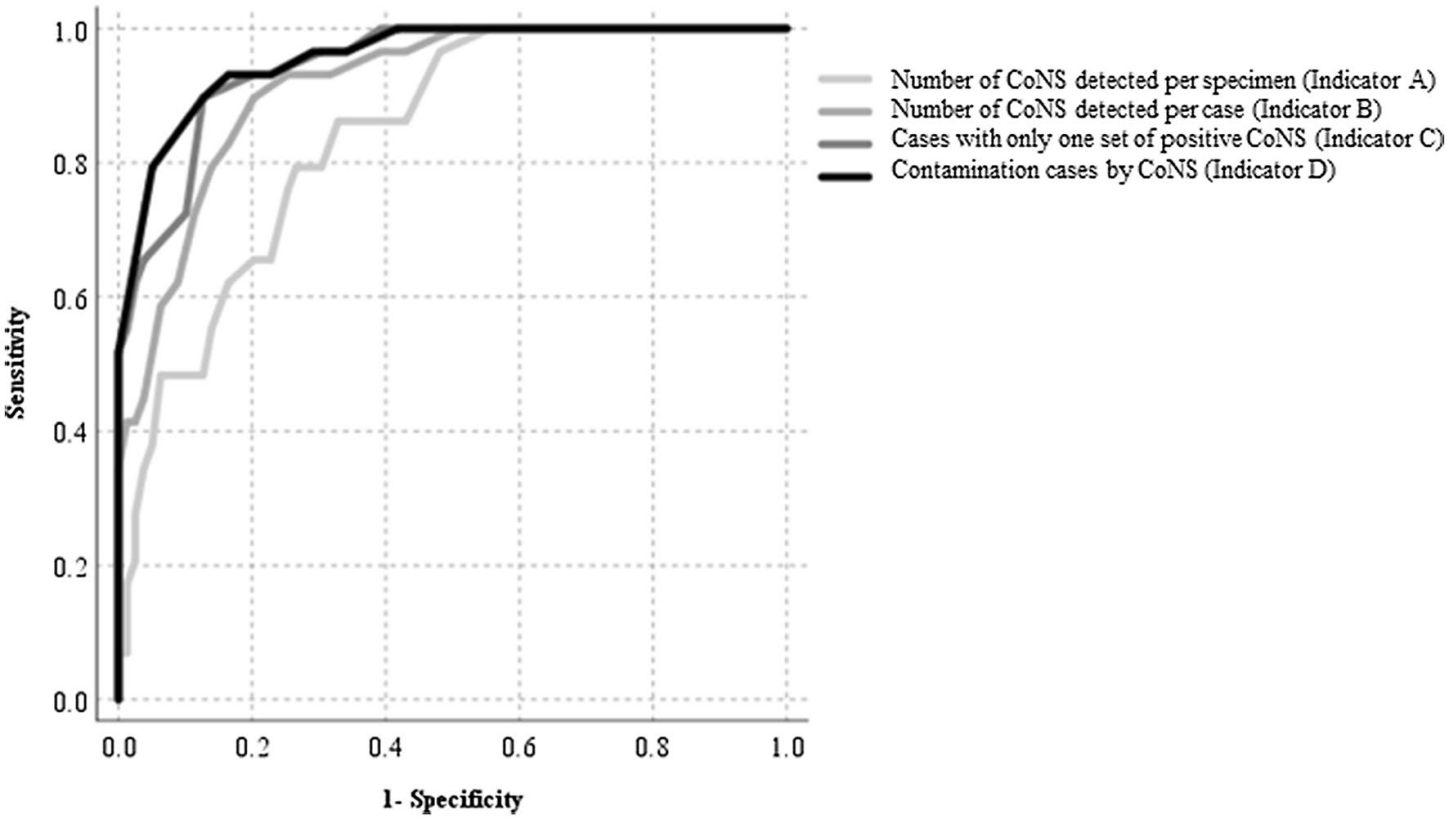
ROC curve predicting ConR 2.5 or higher and AUC comparison. *CoNS* coagulase-negative staphylococci, *ConR* contamination rate, *AUC* Area under the curve, *ROC* Receiver operating characteristic.

A total of 103,339 sets of blood cultures were collected during the study period. Of these, 12,670 sets were culture-positive. Of these, 594 cases (778 sets) were excluded, because of pending determination in 190 cases and no determination in 404 cases. Eligible blood cultures were collected from a total of 56,843 cases. Of these, 520 (0.9%) had three or more sets of specimens collected on the same day. CoNS was positive in 3126 sets, of which *Staphylococcus epidermidis* was included in 1771 sets (56.7%). A total of 2142 cases were determined as contaminated; of these, 1689 (78.9%) cases were contaminated with CoNS. The ConR was 2.5% or higher for 29 months of the total study period (26.8%) (Supplementary Fig. 1). The correlation coefficients with ConR for indicators A–D were 0.71, 0.85, 0.91, and 0.92, respectively (Fig. 1). The ROC curve is shown in Fig. 2, with the area under the curves (AUCs) (95% confidence interval) of 0.84 (0.76–0.92), 0.92 (0.86–0.97), 0.95 (0.91–0.99), and 0.96 (0.92–0.99), respectively. When AUCs for each indicator were compared, we found that A vs. B, A vs. C, A vs. D, B vs. C, and B vs. D were statistically significant with Holm correction (Supplementary Table 1). The sensitivity was 86.2%, 89.7%, 89.7%, and 89.7% and the specificities were 67.1%, 79.7%, 87.3%, and 87.3%, with cut-off values of 29, 23, 19, and 19 cases for indicators A–D, respectively; all with a high negative predictive value (95% confidential interval) of 93.0% (83.0–98.1), 95.5% (87.3–99.1), 95.8% (88.3–99.1), and 95.8% (88.3–99.1), respectively. We compared the predictive ability of monthly contamination rate of 2.5 or higher depending on whether the detected organism was *S. epidermidis* or other CoNS for each sample, but there was no significant difference (AUC 0.784 vs. 0.801, p = 0.76, Supplementary Figure).

## Discussion

These results suggest that the indicators A and B, which can be counted simply, correlated with ConR to predict a ConR of ≥ 2.5, or higher, but their prediction ability was inferior to that of indicators C and D in multiple comparisons of ROC curves. High negative predictive rates were observed for all indicators, indicating that if the number of cases with CoNS detection or CoNS positivity remained low, it was unlikely that the ConR would increase. The sensitivity and specificity for predicting ConR ≥ 2.5 were higher when indicators C or D were used, the former requires time until another blood culture set collected at the same time is determined to be negative, and the latter requires human resources and time to determine contamination. In contrast, indicators A and B do not require much human resources and can be displayed in real time. Most hospitals calculate ConR once a month; however, approximately 30% of the facilities calculate it over a longer time span^4^. Indicators A and B could be good predictors for ConR in such institutes.

ConR of ≤ 3.0 is often used as a standard for the quality of blood culture tests^5^. In this study, we set the predicted ConR to be ≥ 2.5. When the cut-off was increased, the predictive power of each index increased, because the number of months covered decreased; however, there was no difference in the trend of AUC between 2.5% or higher and 3.0% or higher (Supplementary Table 2). ConR is related to the disinfection of the puncture site, collection method, hand hygiene, education, and feedback methods regarding collection^6^. Recently, the usefulness of a blood collection device was reported^7^. These relevant factors can be reviewed when CoNS-positive cases increase. In addition, feedback from monitoring the results alone can improve the ConR^6,8^. Therefore, establishing a system that provides real-time feedback on the number of cases of CoNS detection could be a countermeasure to reduce contamination without requiring additional labour. However, the situation regarding blood cultures varies from hospital to hospital^4^. For example, in facilities with many patients with central venous catheters, true infection by CoNS is more common. In addition, the target ConR varies; and therefore, the results should not be applied directly to facilities that have not set a ConR target of 2.5 or higher. The results of this study, especially the cut-off values, may not be directly applicable to other facilities. However, as CoNS is the most commonly detected organism in contamination, a similar result can be predicted if a cut-off is set and the correlation with ConR at least once at one’s own institution is determined before using it as a simple indicator.

## Methods

### Study design

We performed a retrospective study of patients, who underwent blood culture testing at the National Center for Global Health and Medicine between April 2012 and March 2021. The need for informed consent was waived, because of the retrospective nature of the study design. The study information was presented on the Web for the possibility of opting out of consent. This was substituted for the participants’ consent. The protocol of this study including the opt-out consent method was approved by the Certificate Review Board of National Center for Global Health and Medicine (NCGM-G-004168-00) and conformed to the amended Declaration of Helsinki. The data were compiled from the registry of blood culture surveillance, including data on contamination, and the microbiology laboratory.

### Identification of bacterial species

All blood culture samples were collected into standard aerobic and anaerobic culture bottles (92F or 94F and 93F, 23F or 20F and 24F Becton Dickinson Microbiology Systems, Sparks, MD, USA) and processed using the BACTEC 9240, 9120, and FX systems (Becton, Dickinson and Company, Franklin Lakes, NJ, USA). These samples were routinely monitored for at least 144 h. The bottles that tested positive were removed and subjected to Gram staining. The specimens were then inoculated into 5% sheep blood agar and BTB agar media (Nissui Pharmaceutical Co., Ltd., Tokyo, Japan), and incubated at 35 °C (Depending on the situation, other media may be added, or the environment may be changed, such as anaerobic incubation). Conventional bacterial identification and susceptibilities to the predefined antimicrobials were determined in accordance with the Clinical and Laboratory Standard Institutions criteria (M100)^9,10^ using matrix assisted laser desorption/ionization-time of flight mass spectrometry system (MALDI Biotyper system; Bruker, Billerica, MA, USA) and automated broth micro dilution system (MicroScan WalkAway 96 SI system; Beckman Coulter, Brea, CA, USA). All Staphylococci species, except *S. aureus* and *S. lugdunensis*, were treated as CoNS.

### Blood sampling

The standard procedure recommended that the puncture site be cleaned with an alcohol swab, disinfected twice with poppidone-iodine, and then puncturing a vein or artery to collect a blood specimen. It was recommended to collect blood on two or more different sites. The use of blood collection during catheterization and catheter regurgitation is not recommended, but was allowed. The recommended collection volume is 10 mL of blood per bottle for adult bottles (92F and 93F, 23F and 24F) and 1–3 mL per bottle for paediatric bottles (94F and 20F). Compliance with disinfection methods and sample collection volumes was not monitored.

### Definition of contamination

Contamination was defined as the presence of either one of the two or more sets of skin-resident bacteria (CoNS, *Bacillus* spp. excluding *B. anthracis*, *Corynebacterium* spp., *Cutibacterium* spp., *Streptococcus* Viridans group, *Aerococcus* spp., *Micrococcus* spp.) listed in the CUMITECH^3^. However, even in cases where these bacteria were detected, if a central venous catheter or an intravascular device showed obvious signs of infection such as redness, fever, pus drainage, or pus accumulation, and the patient's condition clearly improved with the administration of a susceptible antimicrobial agent, these cases were not be treated as contamination. In case of enterococci and *Clostridium* spp., if only one set was positive out of two or more sets collected, and there was no obvious foci of infection and the symptoms improved without administration of a susceptible antimicrobial agent, it was considered a contamination. These decisions were made retrospectively, based on the medical records, by infectious disease specialists who perform blood culture surveillance.

### The registry data of blood culture surveillance

For every case, two or more infectious disease physicians of the National Center for Global Health and Medicine determined whether the case was contaminated from a clinical point of view, by reviewing clinical records and laboratory data in accordance with the aforementioned criteria. Undetermined cases and those with pending determination were excluded from the study.

### Indicators

We calculated the monthly ConR [(total number of contaminated cases per month)/(total number of blood culture sets collected per month) × 100]^3^. The values of the four indicators were aggregated as follows: the number of CoNS-positive sets of blood culture (Indicator A), cases with at least one CoNS-positive blood culture set (Indicator B), cases with only one CoNS-positive blood culture set (Indicator C), and cases of contamination by CoNS (Indicator D).

### Statistical analysis

Correlation coefficients were calculated using Pearson’s correlation test. Receiver operating characteristic (ROC) curves were prepared for all indicators with a ConR of ≥ 2.5, as the objective variable, and cut-off values were calculated using Youden's index. The AUCs were compared using the Delong method with the Holm correlation. Statistical analysis was performed using EZR for Windows version 1.54^11^. Figures were created using IBM SPSS Statistics software for Windows (version 26.0; IBM Corp., Armonk, NY, USA). The probability of significance was calculated to be 5%.

### Ethics approval

The need for informed consent was waived due to the retrospective nature of the study design. The study information was presented on the Web for the possibility of opting out of consent. The protocol was approved by the institutional review board of the National Center for Global Health and Medicine (NCGM-G-004168-00).

### Consent to participate

The study information was presented on the Web for the possibility of opting out of consent.

## Supplementary Information

Supplementary Figure 1.

Supplementary Figure 2.

Supplementary Tables.
