# Supplementary figures and images for: Simple indictor of increased blood culture contamination rate by detection of coagulase-negative staphylococci
Source: Sci Rep. 2021 Sep 2;11:17538. doi: 10.1038/s41598-021-96997-y (PMC8413347; doi:10.1038/s41598-021-96997-y)

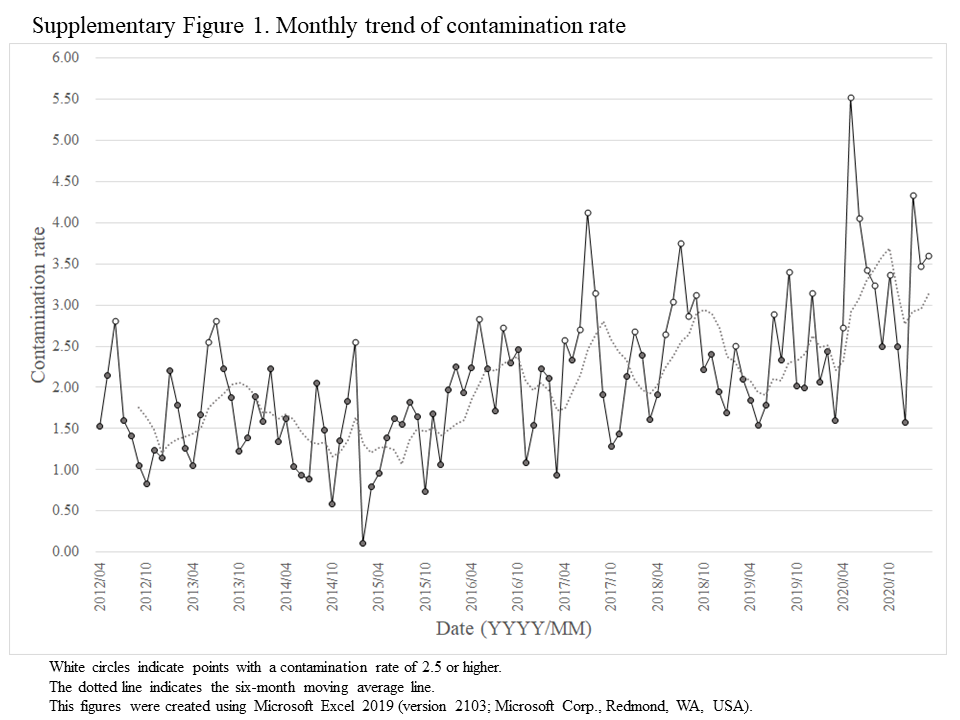

Supplement: Supplementary file 1 — Supplementary Figure 1. [file 41598_2021_96997_MOESM1_ESM.tif]

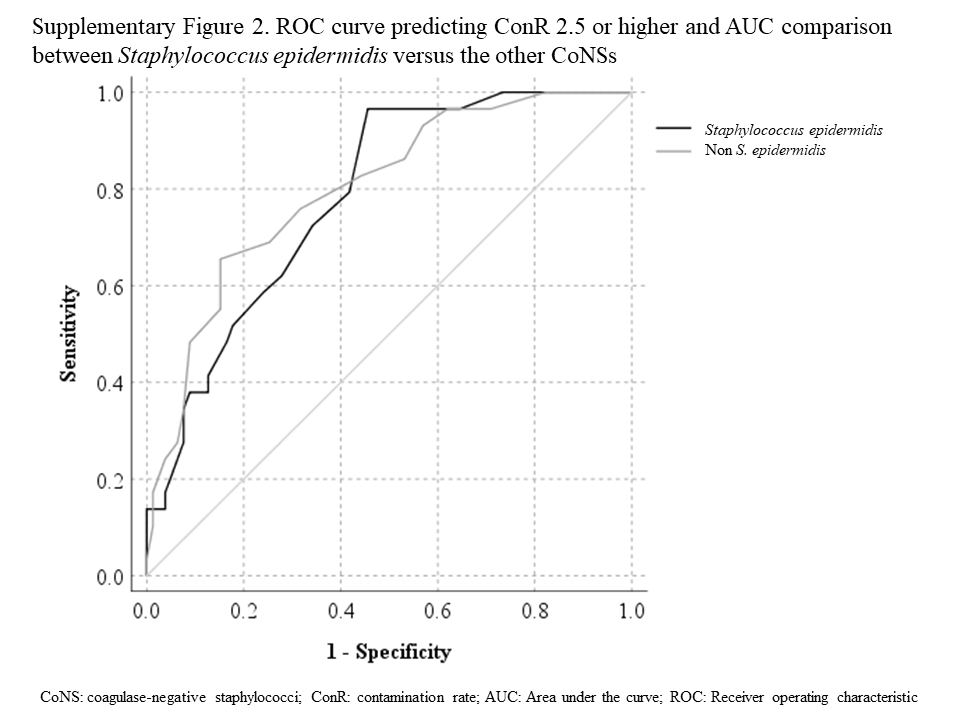

Supplement: Supplementary file 2 — Supplementary Figure 2. [file 41598_2021_96997_MOESM2_ESM.tif]
