# Supplementary material for: Simple indictor of increased blood culture contamination rate by detection of coagulase-negative staphylococci
Source: Sci Rep. 2021 Sep 2;11:17538. doi: 10.1038/s41598-021-96997-y (PMC8413347; doi:10.1038/s41598-021-96997-y)
Supplement: Supplementary file 3 — Supplementary Tables. [file 41598_2021_96997_MOESM3_ESM.pdf]

Simple indictor of increased blood culture contamination rate by detection of coagulase-negative staphylococci

Kei Yamamoto[a*], Kazuhisa Mezaki[b], Norio Ohmagari[a]

Supplementary Table 1. The result of multiple comparisons of the area under the curve

	Indicator A	Indicator B	Indicator C
Indicator B	*p*=0.00074*	-	-
Indicator C	*p*=0.00035*	*p*=0.024*	-
Indicator D	*p*=0.00017*	*p*=0.012*	*p*=0.27

Number of CoNS detected per specimen (Indicator A)
Number of CoNS detected per case (Indicator B)
Cases with only one set of positive CoNS (Indicator C)
Contamination cases by CoNS (Indicator D)
*Those with a significant difference at 5% significance probability by Holm correction.
CoNS: coagulase-negative staphylococci

Supplementary Table 2. The area under the curve calculated from the ROC curve when the blood culture contamination rate to be predicted was changed.

Condition of contamination rate	Months that meet the conditions, n (%)	Indicator A	Indicator B	Indicator C	Indicator D
		Area under the carve			
2.0	53	0.923	0.963	0.981	0.981
2.5	29	0.840	0.916	0.945	0.957
3.0	15	0.891	0.933	0.969	0.958
3.5	6	0.910	0.938	0.962	0.972
4.0	3	0.941	0.972	0.977	0.987

Number of CoNS detected per specimen (Indicator A)
Number of CoNS detected per case (Indicator B)

Cases with only one set of positive CoNS (Indicator C)
Contamination cases by CoNS (Indicator D)
CoNS: coagulase-negative staphylococci; ROC: Receiver operating characteristic
